# Nurses’ Perception of Caring and Attitudes toward Caring for Dying Patients: Exploring the Relationship Using a Descriptive Cross-Sectional Design

**DOI:** 10.3390/healthcare12131331

**Published:** 2024-07-03

**Authors:** María del Carmen Fernández-Gutiérrez, Isabel Benavente-Fernández, Natalia Jiménez-Luque, Simón Lubián-López, Consuelo López-Fernández, Juan Manuel Picardo-García

**Affiliations:** 1 Critical Care Unit, Puerta del Mar University Hospital, 11009 Cádiz, Spain; mcfdez15@gmail.com; 2 Biomedical Research and Innovation Institute of Cádiz (INiBICA) Research Unit, Puerta del Mar University, 11009 Cádiz, Spain; isabel.benavente@uca.es (I.B.-F.); simonplubian@gmail.com (S.L.-L.); 3 Area of Pediatrics, Department of Child and Mother Health and Radiology, Medical School, University of Cádiz, 11003 Cádiz, Spain; 4 Division of Neonatology, Department of Pediatrics, Puerta del Mar University Hospital, 11009 Cádiz, Spain; 5 Department of Nursing, School of Nursing and Physiotherapy, University of Cadiz, 11009 Cádiz, Spain; consuelo.lopez@gm.uca.es (C.L.-F.); juan.picardo@gm.uca.es (J.M.P.-G.)

**Keywords:** nursing, end-of-life care, caring, attitude, health, death

## Abstract

This study examines the association between nurses’ perception of caring and attitudes toward caring for dying patients in end-of-life care units. We used a descriptive cross-sectional design with convenience sampling. Data were collected from May to June 2021 through self-reported questionnaires from 303 Spanish nurses (mean age = 48 years, 78.9% female). Participants completed the Caring Dimension Inventory (CDI-25) and the Frommelt Attitudes Toward Care of the Dying scale. Demographic and training information were collected. Data were analyzed using Stata 16.0. Attitudes toward Care of the Dying Patient were significantly higher in nurses with palliative care training (β = 2.829, *p* = 0.018) and those who scored higher on the psychosocial (β = 0.789, *p* = 0.0001) and technical dimensions (β = 0.487, *p* = 0.011) of the CDI-25. Multivariate analysis identified the psychosocial dimension of the CDI-25 scale (β = 0.783, *p* = 0.0001) and palliative care training (β = 2.686, *p* = 0.017) as the only significant variables associated with Attitudes toward Care of the Dying Patient. Overall, nurses exhibited a positive attitude toward caring for dying patients. Our results could potentially help nursing directors identify those with the necessary qualities and training for optimal end-of-life care and to further plan training courses to provide optimal care in end-of-life situations.

## 1. Introduction

While home care for dying patients is becoming more common, the majority of care for these patients is still provided by nurses in general hospital wards [1]. Therefore, the professional role of nurses often brings them into close contact with patients and families of dying patients, and they must be able to manage and respond appropriately to the suffering this entails [2].

The care of dying patients poses significant challenges that require both advanced skills and appropriate attitudes. The nursing skills required to provide optimal care for these patients encompass various dimensions, including biological, psychological, social, and spiritual aspects [3]. The attitudes of nurses towards death can greatly influence their approach to caring for dying patients and impact their professional behavior [4]. And, on the other hand, nurses’ positive perceptions of death, and their attitude towards caring for the dying patient and their family, are significantly associated with better performance in caring for these patients [5]. Nurses should establish intervention strategies for the effective accompaniment of family members of dying patients [6].

It has been found that nurses’ attitudes toward caring for the dying patient are connected to several sociodemographic, personal, and training factors: gender [7], number of years spent working as a registered nurse [8], having worked with terminally ill patients [9], personal attitudes toward death [10], having experienced the death of a family member or other close individual [11], work engagement [12] or nurses’ autonomy [13].

Although caring is a very important concept in nursing, that encompasses a range of behaviors, practices, and attitudes aimed at promoting the well-being and comfort of patients [14], to date, its relationship with attitudes toward caring for dying patients has not been studied, probably because it is difficult to assess [15]. Based on this, a number of tools have been developed to measure nursing care [16,17]. Most of these instruments are designed based on sound theoretical frameworks: Mayeroff’s eight caring ingredients (Caring Ability Inventory [18], Watson’s theory of human caring (Caring Behavior Inventory) [19], or a combination of different theories (Caring Attributes, Professional Self, and Technological Influence Instrument) [20] or the Caring Dimension Inventory (CDI) [21]. CDI is one of the most frequently used instruments when measuring caring [16,17] and is applicable to nurses from different cultures [22,23,24].

The present study was designed to examine the association between nurses’ perceptions of care and their attitudes toward caring for dying patients among nurses who had worked in units providing such care. Thus, this study aims to analyze the relationship between demographic characteristics (age, gender, marital status, years of experience, etc.) and perceptions of care and attitudes toward caring for dying patients.

## 2. Materials and Methods

### 2.1. Study Design and Sample

This study adopted a descriptive cross-sectional research design using convenience sampling on a total of 303 nurses who were recruited from Puerta del Mar University Hospital in Cádiz (Spain). The inclusion criteria were: (1) full-time registered nurses who had worked in units where there had been more than 5 deaths in the previous year and (2) verbal informed consent and voluntary participation in the study. The exclusion criteria involved newly employed nurses who were trainees under the supervision of preceptors or nurse managers. The sample size was estimated by using Stata version 16.0. According to previous studies using FATCOD, we considered a standard deviation of 10 and we estimated the sample size that would allow detecting a minimum effect size of 5, which was considered clinically relevant [25]. A sample size of 170 participants was estimated necessary to achieve this comparison with an alpha risk of 0.05, and a power level of 0.90.

### 2.2. Variables

#### 2.2.1. General Information Questionnaire

General information of the participants was collected through a self-reported questionnaire that included information about gender, age, marital status, children, religious beliefs, years of work experience, type of nursing unit, palliative care education and whether the participant had ever experienced the death of immediate family members.

#### 2.2.2. Caring Dimension Inventory (CDI-25)

CDI-25 is a 25-item, 5-point Likert scale designed to measure nurses’ perception of caring [21]. Participants rate each item on the scale ranging from 1 (strongly disagree) to 5 (strongly agree). The tool includes five dimensions: psychosocial (10 items), physical-technical (11 items), professional (1 item), unnecessary (1 item) and inappropriate (2 items). Items 3 and 16 are scored in reverse, so that the strongly agree and strongly disagree options are given the lowest and highest scores, respectively. Scoring is accomplished by summing scores for items. The total score ranges from 25–125. A higher score suggests that nurses perceive the caring aspects of their profession to be more focused on professional and technical elements. Conversely, lower scores indicate that nurses primarily perceive caring to be associated with psychosocial aspects. The score solely reflects the perception of caring in relation to these aspects and should not be interpreted as a direct indicator of the extent of an individual nurse’s care. While the original scale demonstrated a Cronbach’s alpha of 0.91, in this sample, the Cronbach’s alpha for the total scale was 0.77.

#### 2.2.3. Frommelt Attitudes toward Care of the Dying (FATCOD) 

FATCOD scale assess individuals’ attitudes toward providing care to dying patients [26]. The scale consists of 30 items, divided into positive and negative questions, each with 15 items. The scale includes 2 dimensions: the nurses’ attitudes toward the dying patient and toward family members. A Likert 5-point scale is used to assign 1–5 points for each positive item, while negative items are assigned points in reverse, for the total score ranges from 30–150. Higher scores indicate more positive attitudes toward providing care for dying patients. The FATCOD has demonstrated high reliability and validity. Its internal reliability is high, typically ranging from Cronbach’s alpha = 0.805 to 0.860, and the test-retest reliability is 0.71. In this sample, the Cronbach’s alpha was 0.81.

### 2.3. Statistical Methods

Categorical variables are expressed in frequency (percentage). Quantitative variables are expressed as mean (SD) or median [IQR] depending on their distribution. Linear regression models were used to study the relationship between the studied variables and total FATCOD score. Multivariate regression was performed including variables selected based on the theoretical background and a backward stepwise approach was performed to exclude the variables not significant if they were not considered variables needed to adjust for. Statistical analysis was conducted using Stata 16.0. A result was considered statistically significant at *p* < 0.05.

### 2.4. Ethical Consideration and Data Collection

The institutional research board in the first authors’ institution approved this study protocol. This study was conducted in accordance with the Declaration of Helsinki. Data were collected after obtaining permission from the chief of nursing administrator of the hospital from 1 May to 30 June 2021. During the research project, a researcher conducted visits to each unit during shift changes to deliver a comprehensive briefing on the study’s objectives, methodology, and ethical considerations. Subsequently, questionnaires were distributed to nurses who voluntarily agreed to participate. All eligible nurses received an information sheet that provided a detailed overview of the study, including their rights regarding participation and withdrawal. To guarantee participant anonymity and confidentiality, completed questionnaires were carefully sealed in envelopes prior to collection. All participants completed the questionnaires anonymously, with a unique identification number (ID) assigned to each participant. Physical copies of the surveys were securely stored in a locked cabinet and will be securely disposed of within a five-year timeframe after the study’s completion. Study data were managed using REDCap electronic data capture tools hosted at Northwestern University [27].

## 3. Results

### 3.1. Demographic Characteristics

A total of 306 nurses, 84% of the nurses in the units studied, participated in this survey and 303 valid questionnaires were collected, an effective recovery rate of 99.01%. Of the 303 participants, 239 (78,9%) were female. The median age of the participants was 48 years [interquartile range (IQR) 41–54] and the median number of years in clinical practice was 19 [IQR 8–30]. More than half of the participants (60.7%) were married and 70.6% had children. 76.8% had religious believes, most of them Catholics.

The number of years dedicated to clinical practice was 19.1 (SD 11.4) years. Half of the participants (54.5%) had palliative care education (61.7%), and 107 (35.7%) nurses had experienced the loss of a family member in the past year.

### 3.2. Descriptive Scores on Assessment Tools

The medians on the different dimensions of the Caring Dimension Inventory (CDI-25) were: psychosocial dimension 55 [IQR = 52–58]; technical 35 [IQR = 33–37]; appropriate 6 [IQR = 4–7]; unnecessary 5 [IQR = 4–6]. The mean score on the Attitudes toward Care of the Dying Patient (FATCOD) scale was 117.8 (±SD 10.4). See Table 1.

### 3.3. Univariate Regression Analysis

As shown in Table 2, age, gender, marital status, having children, religious beliefs, experience of death of a family member or type of unit (ward) were not associated with nurses’ attitudes toward providing care to dying patients. Scores on nurses’ attitudes towards care of dying patients were higher for those with palliative care training (β = 2.829; *p* = 0.018). The mean FATCOD score in nurses who had received palliative care training was 119.10 (±10.7), while in those who had not received training it was 116.27 (±9.7); *p* = 0.009.

Psychosocial (β = 0.789; *p* = 0.0001) and technical (β = 0.487; *p* = 0.011) dimensions of the CDI-25 scale are associated with Attitudes toward Care of the Dying Patient (FATCOD).

### 3.4. Multivariate Analysis of Attitudes toward Hospice Care

When exploring the association of different variables to Attitudes toward Care of the Dying Patient in a multivariate model we found that the psychosocial dimension (β = 0.783; *p* = 0.0001) of the CDI-25 scale and palliative care training (β = 2.686; *p* = 0.017) were the only characteristics that remained significant in relation with attitude toward care of the dying patient. When examining the standardized β coefficients the psychosocial dimension of the CDI-25 scale (βst 0.34) had an association with Attitudes toward Care of the Dying Patient that was greater than palliative care training (βst 0.13).

Age and sex remained non-significant and technical dimension of the CDI-25 lost its significance when included in a multivariate model. Parameters of the selected model are summarized Table 3.

## 4. Discussion

The purpose of this study was to examine the relationship between nurses’ perception of caring and their attitudes toward providing care to dying patients in the context of providing end-of-life care. Our findings shows that the psychosocial dimension of the Caring dimension inventory (CDI-25) scale, designed to measure nurses’ perception of caring, and, to a lesser degree, palliative care training, are associated with more positive attitudes toward providing care for dying patients. To our knowledge, it has not been studied whether nurses’ perception of caring is related to attitudes toward providing care for dying patients.

Care is a fundamental component of nursing practice. The perception of care by nurses shapes their overall view of the profession and their approach to patient care. The fact that, of the four dimensions assessed in the CDI-25 (psychosocial, technical, appropriate and unnecessary), the psychosocial dimension is the one associated with better attitudes toward care of the dying patient is consistent with the previously described relationship between certain psychological characteristics and nurses’ attitudes toward care of the dying patient. Previous studies have found that higher levels of empathy are associated with more positive attitudes toward palliative care [28]; and nurses’ fear of death and death-avoidance attitudes negatively affected attitudes toward caring for terminally ill patients [29,30]. Thus, our results are in line with these findings in such a way that those nurses who perceived their care with a more psychosocial dimension had a better attitude towards the care of the dying patient.

Most nurses in the current study expressed positive attitudes toward caring for dying patients. The overall FATCOD score, mean 117.8 (±SD 10.4), was higher than that found in studies by Karadag et al. (Mean ± SD, 97.19 ± 8.99) [31] and Lancaster et al. (Mean ± SD, 101.45 ± 15.99) [32] and similar to that reported by Ho et al. (Mean ± SD, 116.8 ± 11.4) [33]. The latter study was carried out in the same country as the present one, so the differences between our results and those found in studies carried out in other countries may be explained by cultural variations between the countries studied, such as religious beliefs or differences in the health care system.

The exploration of the contrasting attitudes among nurses concerning end-of-life (EOL) care is a subject that necessitates appropriate attention and scrutiny. Some studies suggest that age may play a significant role in determining one’s outlook towards death and dying [8]. Several studies have indicated that older nurses tend to exhibit more favorable attitudes when it comes to providing care for patients nearing the end of life [12,34]. And this could be because older, more experienced nurses may have a greater understanding of life due to their rich life experiences and, consequently, be better equipped to empathize with dying patients and communicate empathetically with their families [8,34,35,36]. Our results do not support these findings, we found no relationship between age or years working as a nurse and attitude towards the care of the dying patient. The discordance of our results may be due to the fact that the age of the nurses participating in our study was older than that of the other studies, so the effect of this variable on the attitude towards the care of the dying patient may be mitigated.

Some studies find differences in nurses’ attitudes towards the care of the dying patient according to the department in which they work [37]. Our findings do not confirm these results possibly because in our study we have included nurses working in departments with a minimum number of patients per year in EOL situation.

Years of work experience [9,33] and previous training in palliative care [38,39,40,41] have been associated with more positive attitudes towards the dying patient. As in these studies, our study shows that palliative care training leads to more positive attitudes when working with the dying patient and family members. Palliative care training could help to positively change nurses’ attitudes toward caring for dying patients and their families [42].

### Strengths, Weaknesses, and Limitations

The main outcome of this study was measured with a well-known research instrument. This tool was used in a geographic area where nurses’ attitudes toward caring for dying patients had not previously been studied. Our results indicated that the tool demonstrated good internal consistency.

This study has several weaknesses that must be taken into consideration. Firstly, it was conducted exclusively within a single hospital, thereby posing challenges in generalizing the findings to other healthcare facilities and nurse populations. Cultural and population variations in perceptions of death and attitudes towards it, influenced by individual values and beliefs, make it arduous to extrapolate these results to different contexts. Secondly, although the study achieved a high response rate, there were three invalid questionnaires due to lack of full completion, and the characteristics of non-respondents were not established, which could introduce sampling biases and limit the representativeness of the sample. Among the limitations is the reliance on self-reported questionnaires as the sole data collection method may introduce inaccuracies in capturing participants’ attitudes towards end-of-life care. Furthermore, despite the sufficient sample size, the study analyzed the results of two scales; however, more variables involving nursing care should be considered. These weaknesses and limitations underscore the importance of further research that accounts for these factors to enhance the precision of findings and promote the provision of effective care of the dying patient.

## 5. Conclusions

The study revealed significant positive correlations between nurses’ perceptions of care, and training in palliative care, and their attitudes toward caring for dying patients in acute care hospital settings. Training in palliative care appears to enhance nurses’ perspectives on caring for dying patients. This is crucial because every nurse in the healthcare field may face end-of-life situations and need to support dying patients and their families. On the other hand, nurses who perceive their care with a more psychosocial dimension have more positive attitudes towards the care of dying patients, demonstrating that better care for these patients requires nurses with certain qualities. These findings may assist nursing directors in the selection of nurses with a profile best suited to care for the dying patient. Future research is needed to extrapolate our results to other cultural settings with organizations other than the health care system and to study the factors that are associated with positive attitudes toward caregiving, not only of the patient, but also of the families of the dying patient.

## Figures and Tables

**Table 1:** Characteristics of the study population.

Variable ^1^		N = 303
Age (years)		47.5 (8.8)
Sex (female)		239 (78.9%)
Married		184 (60.7%)
Children		214 (70.6%)
Religious beliefs		233 (76.8%)
Nursing experience (years)	1–10	92 (30.4%)
	10–20	77 (25.4%)
	20–30	74 (24.4%)
	>30	60 (19.8%)
Ward	Critical care	83 (27.3%)
	Internal medicine	64 (21.1%)
	Emergency	58 (19.1%)
	Surgical area	52 (17.1%)
	Paediatrics	34 (11.2%)
	Oncology	12 (3.9%)
Palliative care education		165 (54.5%)
Experience of death of a family member (within last year)		107 (35.7%)
Caring dimension inventory (CDI-25)	Psychosocial dimension	55 [52–58]
	Technical dimension	35 [33–37]
	Appropriate dimension	6 [4–7]
	Unnecessary dimension	5 [4–6]
Frommelt Attitudes Toward Care of the Dying (FATCOD)		117.8 (±SD 10.4)

^1^ Categorical variables are expressed in frequency (percentage). Quantitative variables are expressed as mean (SD) or median [IQR] depending on their distribution.

**Table 2:** Association of socio-demographic characteristics and nurses’ perception of caring scores to participants ‘attitudes toward providing care to dying patients.

Variable ^1^		Β Coeff	*p* Value
Age (years)		−0.076	0.265
Sex (female)		1.418	0.332
Married		−0.591	0.673
Children		0.911	0.488
Religious beliefs		0.135	0.878
Nursing experience (years)	1–10	Ref	Ref
	10–20	0.896	0.577
	20–30	−0.619	0.704
	>30	−1.051	0.542
Ward	Critical care	1.593	0.132
	Internal medicine	−8.277	0.129
	Emergency	1.593	0.132
	Surgical area	−1.115	0.060
	Paediatrics	−0.467	0.739
	Oncology	−9.667	0.148
Palliative care education		2.829	0.018
Experience of death of a family member (within last year)		0.632	0.615
Caring dimension inventory (CDI-25)	Psychosocial dimension	0.789	0.0001
	Technical dimension	0.487	0.011
	Appropriate dimension	0.385	0.300
	Unnecessary dimension	0.196	0.602

^1^ Β coeff = β coefficient. The bivariate analysis was performed with simple lineal regression to study the association of each variable to FATCOD score.

**Table 3:** Multivariate linear regression model showing the association of the psychosocial dimension of the CDI-25 scale and palliative care training with participants ‘attitudes toward providing care to dying patients (FATCOD).

Variable	FATCOD	
	Beta	*p*
Palliative care education	2.686	0.017
Psychosocial dimension (CDI-25)	0.783	0.0001

## Data Availability

Data are contained within the article.
